# Supplementary material for: Activity of Mono-, Bi-, and Trimetallic Catalysts Pt-Ni-Cr/C in the Bicyclohexyl Dehydrogenation Reaction
Source: Molecules. 2022 Dec 1;27(23):8416. doi: 10.3390/molecules27238416 (PMC9737465; doi:10.3390/molecules27238416)
Supplement: Supplementary file 1 [file molecules-27-08416-s001.zip › molecules-1991950-supplementary.pdf]

Supplementary Materials

Activity of Mono-, Bi-, and Trimetallic Catalysts Pt-Ni-Cr/C in the Bicyclohexyl Dehydrogenation Reaction

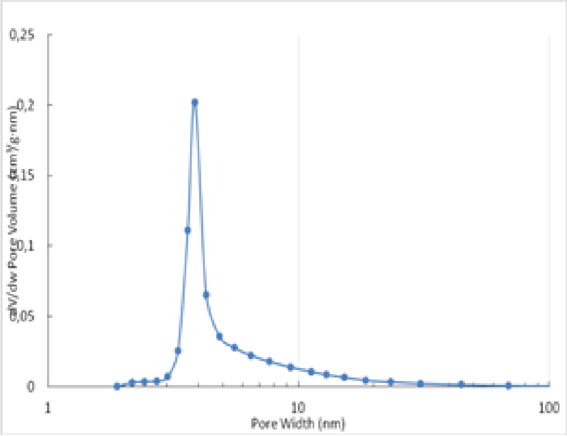

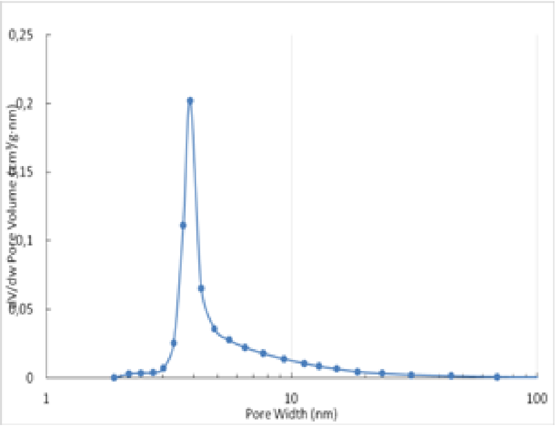

(**a**) (**b**)

Figure S1. Pore size distributions for Sibunit before (a, C) and after oxidation (b, C_{ox}).

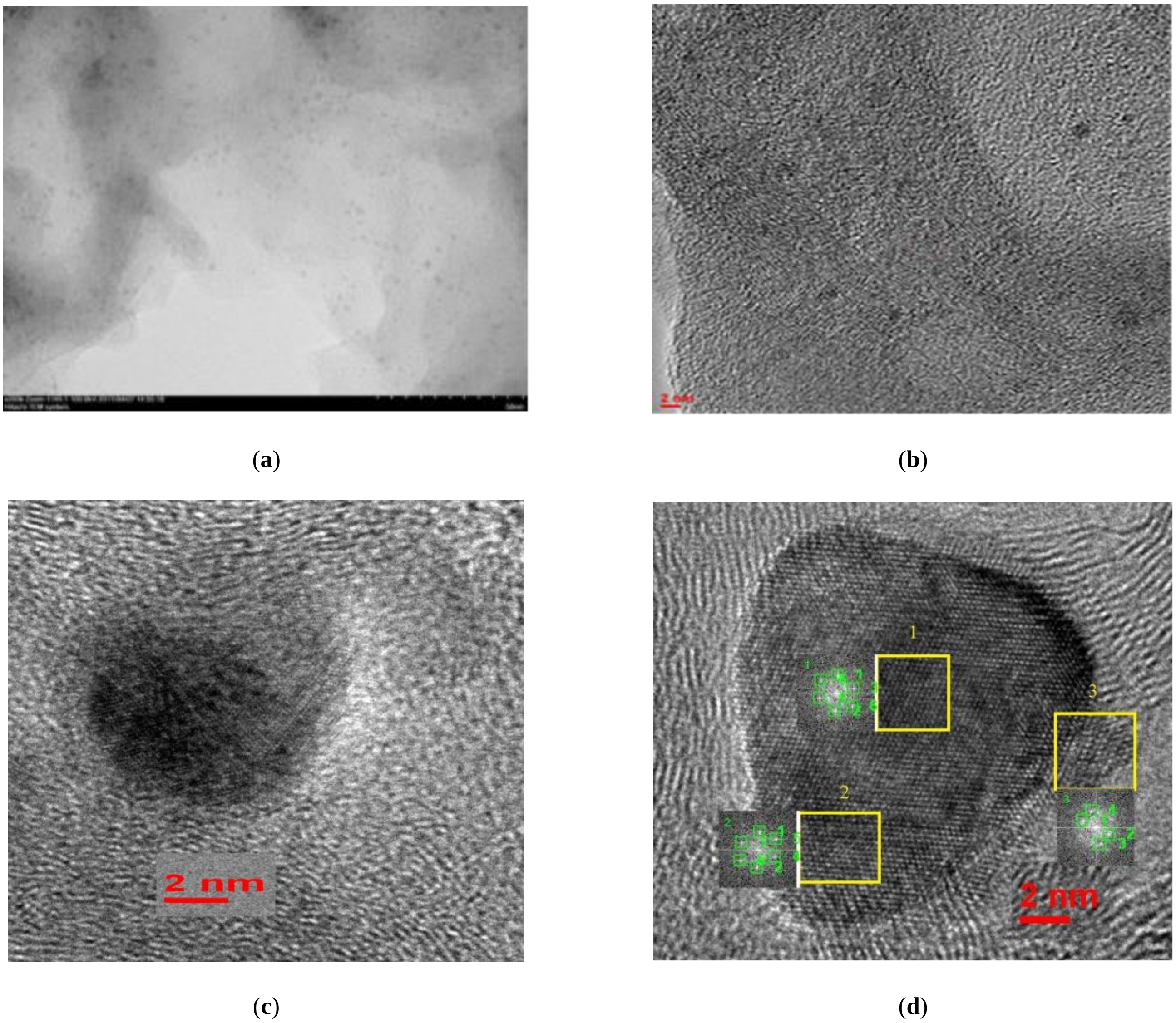

Figure S2. TEM images of catalysts 0.1Pt/C (a), 0.1Pt/1.5Cr/C (b), 0.1Pt/3Ni/C (c), 0.1Pt/1.5Cr/3Ni/C (d).

Table S1. XPS data for the catalysts before and after reduction in H_2.

Catalyst	Pt 4f				Ni2p				Cr2p	
	E, eV	%	*E*, eV	%	*E*, eV	%	*E*, eV	%	*E*, eV	%
0.1Pt/C	71.2	21	72.0	79	-	-	-	-	-	-
3Ni/C	-	-	-	-	852.5	53	855.8	47	-	-
0.1Pt/3Ni/C	71.2	91	72.0	9	852.5	32	855.8	68	-	-
0.1Pt(3Ni-1.5Cr)/C	71.2	49	72.0	51	852.5	38	855.8	62	577.0	100
0.1Pt/1.5Cr/3Ni/C	71.2	33	72.0	67	852.5	39	855.8	61	577.0	100

Table S2. Temperatures of the maxima at the TPR curves for the studied catalysts.

Catalyst	Temperatures of hydrogen uptake		
	T = 50–270°C	T = 300–450°C	T= 450–850°C
C (Sibunit)	-	-	680
0.1Pt/C	-	-	640
3Pt/C	270	-	510
3Ni/C	190	330	540
0.1Pt/3Ni/C	270	340	-
0.1Pt/(3Ni-1.5Cr)/C	171	337	539

Table S3. Magnetic properties of the PtNiCr/C catalysts.

Catalyst	Concentration of ferromagnetic Ni, C_m wt.%		Size of Ni particles, *d*, nm	Curie temperature, T_C, °C
	Starting	After treatment in H_2		
3Ni/C_{ox}	0.3	1	-	350
0.1Pt/3Ni/1.5Cr/C_{ox}	0	0.7	5–12	342
0.1Pt/1.5Cr/3Ni/C_{ox}	1.5	2.2	6–17	323
0.1Pt/(3Ni-1.5Cr)/C_{ox}	0.9	1.5	7–12	343
